# Droplet digital PCR versus real-time PCR for in-house validation of porcine detection and quantification protocol: An artificial recombinant plasmid approach

**DOI:** 10.1371/journal.pone.0287712

**Published:** 2023-07-14

**Authors:** Umi Nuraeni, Jekmal Malau, Retno Tri Astuti, Auraga Dewantoro, Dini Apriori, Evellin Dewi Lusiana, Bambang Prasetya

**Affiliations:** 1 Laboratory of National Measurement Standards of Biology, The National Standard Agency of Indonesia (BSN), South Tangerang, Banten, Indonesia; 2 Department of Pharmacy, Faculty of Health Science, Universitas Singaperbangsa Karawang, West Java, Indonesia; 3 Department of Fisheries Product Technology, Faculty of Fisheries and Marine Science, Universitas Brawijaya, East Java, Indonesia; 4 Research Center for Genetic Engineering, The National Research and Innovation Agency of Indonesia (BRIN), Bogor, Indonesia; 5 Department of Aquatic Resource Management, Faculty of Fisheries and Marine Science, Universitas Brawijaya, East Java, Indonesia; 6 Research Center for Testing Technology and Standards, The National Research and Innovation Agency of Indonesia (BRIN), South Tangerang, Indonesia; Imam Abdulrahman Bin Faisal University, SAUDI ARABIA

## Abstract

Authenticity and traceability are essential for modern food and medicine inspection, and reliable techniques are important for the trade of halal foods, which reach more than 20 percent of the world market. A sensitive and accurate porcine detection method is required to develop a conformity assessment system that includes laboratory testing for porcine-free certification. This study proposes a procedure that could be incorporated into the development of a standardized control and protocol for real-time PCR (qPCR) methods and their traceability using droplet digital PCR (ddPCR). The design used a recombinant pUC57 plasmid as an amplification target to carry the 97 bp fragment of the porcine ATCB gene. The absolute quantification and linearity assessment showed high precision with R2 values of 0.9971 and 0.9998 for qPCR and ddPCR, respectively. In general, both methods showed comparable results in terms of linearity and detection limit. However, both limit of detection assessments showed high sensitivity, although ddPCR showed a slightly higher sensitivity than that of qPCR, especially at low DNA concentrations. Multiple-sample and inter-participatory testing evaluations revealed a high sensitivity, broad applicability, and robustness of the qPCR method. Therefore, we conclude that based on a recombinant plasmid analysis with a low quantity (less than five copy number), the digital PCR method produced more reliable results. These results could provide scientific information for regulatory authorities, especially those in Indonesia, to consider the development and formulation of a well-established qPCR protocol for porcine detection using expected DNA concentrations.

## Introduction

Food product integrity and authenticity are crucial requirements in the field of legal food regulation [1]. Meat-based products are one of the most highly demanded processed foods that often include a mixture of multiple species [2]. Accurate and detailed information should be provided for these products, including the meat composition and respective percentages [3]. Deceiving customers regarding the quality or composition of unconventional meat mixtures constitutes food fraud. Food fraud can have detrimental effects on customer health (e.g., food allergies and zoonotic disease) and negatively impact wild endangered animal species, consumer beliefs, religious concerns, public trust, and the general economy of the global food industry [4].

Porcine materials, such as meat, lard, and bone extract, are often added to food products because of their price or availability or because they present a desirable taste, flavor, or characterization. Despite its beneficial characteristics, porcine could be a source of food adulteration when added to processed foods without clear labeling. Porcine adulteration and mislabeling can be serious concerns in terms of zoonotic diseases, food allergies, and religious beliefs. Muslims and Jews are prohibited from consuming pork or other pork-containing foods for religious reasons [5, 6]. To protect consumer rights, health, religious beliefs, and public trust, the development of a national standard protocol for porcine detection and quantification is urgently required.

A variety of detection methods have been developed, including protein-based and DNA-based technologies. In general, protein-based methods detect specific proteins or peptides using techniques such as sodium dodecyl-sulfate polyacrylamide gel electrophoresis (SDS-PAGE), enzyme-linked immunosorbent assay (ELISA), spectrometry, and spectroscopy [7–9]. Protein-based methods, such as ELISA, are effective for fresh products and have a relatively low cost and equipment requirement [7]. However, since proteins are easily denatured, this approach is not effective for processed products such as meat patties, canned food, or dried products. DNA-based methods are highly effective for heat-processed food products and different food matrices; therefore, these techniques are stimulating increased interest. Various strategies are applied for mixture detection using DNA-based methods, such as DNA hybridization, DNA barcoding, polymerase chain reaction (PCR), DNA-Foil technology, and isothermal amplification [6, 8, 10].

Among the various DNA-based methods, quantitative polymerase chain reaction (qPCR) is well-established, provides an acceptable level of precision and sensitivity, and is widely used for porcine identification [11–13]. However, this technique relies on quantification cycle (Cq) analysis (which can vary within the instrument and user interpretation), has an uncertain precision at low target DNA levels and high matrix complexities, and requires appropriate reference material and a standard curve for each quantification analysis run [14, 15].

Droplet digital PCR is a recently developed technology that has been reported to provide more accurate DNA quantification. The main advantages of the ddPCR technique are the absolute quantification of specific nucleotide fragments without external references and standard curves [16, 17], a higher precision and sensitivity than those of qPCR [18–20], and a relatively stable result in the presence of reaction inhibitors [21]. This method permits absolute counting based on water-oil emulsion and individual separation. A template-containing sample is diluted into specific points and partitioned into thousands of droplets, and amplification of specific target DNA occurs in each separated droplet, generating fluorescence that is recorded as a positive or negative result for each bubble. This data is then calculated based on specific Poisson algorithms to absolutely quantify the target DNA [22].

Digital PCR technique has been tested for many purposes, such as GMO (genetically modified organism) analysis [23], disease detection (such as for cancer and pathogens) [24], food authentication [25, 26], and SARS_CoV 2 detection [27]. In addition, in-house validation methods for dPCR have been conducted using specific certified reference material [28]. However, the development of ddPCR for porcine detection and validation of widely used qPCR-based methods is still in the exploration stage.

**Fig 1:**
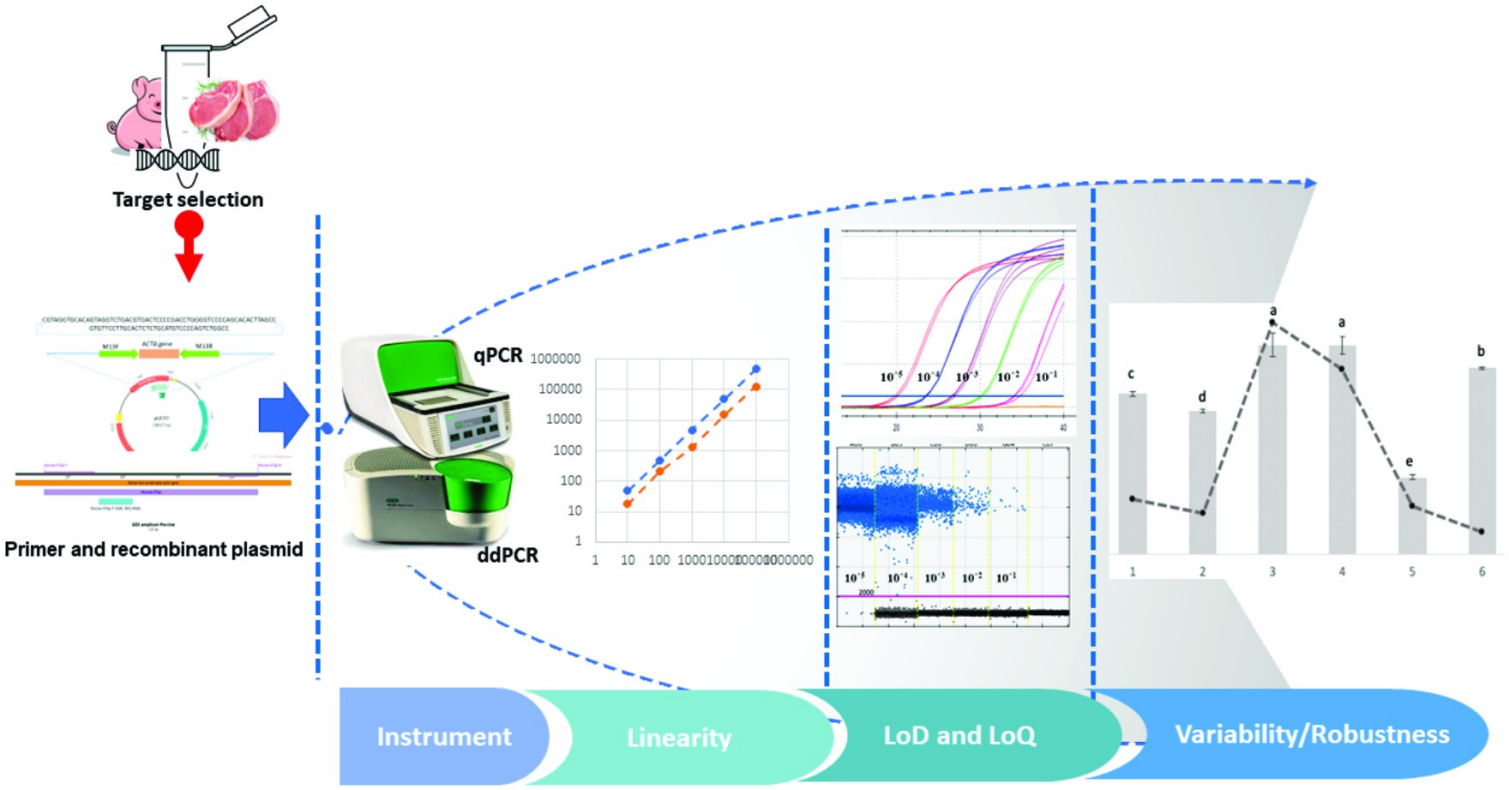
Graphical abstract.

The present study directly compared ddPCR with qPCR to contribute to the development of a validated protocol for detecting and quantifying porcine materials. As opposed to direct sample analysis, an artificial recombinant pUC57 plasmid carrying synthetic a porcine-specific gene was used as the amplification target. A variety of natural target genes, either from mitochondrial DNA (mtDNA) or nuclear DNA (nDNA), have been extensively studied. In general, mtDNA provides a high sensitivity, although it presents with multiple copies and can different types of tissues that could cause quantification biases and reduce measurement accuracies [29]. Thus, the recombinant pUC57 plasmid carrying the ACTB gene, which is an nDNA, was preferred for this study. The designed artificial plasmid enabled the copy number of the target DNA to be calculated, which is crucial for protocol validation under controlled conditions. The determination of the standard plasmid, linearity, and efficiency of the reaction, limit of detection (LOD), limit of quantitation (LOQ), and performance were investigated using various samples (Fig 1).

To the best of our knowledge, this is the first report to directly compare ddPCR and qPCR techniques in a porcine assay based on artificial recombinant plasmid DNA. Data generated from the present study will be important for determining correction factors for both techniques. This research will contribute to the development of an external standard for qPCR-based quantification and a routine inspection protocol for instrument performance and kit and interlaboratory-user capabilities in national-wide inspections. In addition, a scientific consideration will be submitted to regulatory authorities for the development of an Indonesian national standard for porcine-free certification.

## Materials and methods

### Plasmid, primers, and probe

A pUC57 recombinant plasmid was constructed by inserting the porcine-specific DNA fragment using a previously described method [12], with slight modifications. This plasmid was designed as a control to determine the linearity, LOD, and LOQ of the ddPCR and qPCR techniques with gene insertions and was used as a DNA standard in this in-house validation. The plasmid was synthesized by Genescript Biotech (Nanjing, China) (S1 Fig).

A primer-probe set targeting the *Sus scrofa-specific beta-actin* (ACTB) gene was used for porcine detection. The gene bank accession number was DQ452569.1 (NCBI). The primers and Taqman probe sequences are shown in Table 1 [12, 30]. The primers and the probe were synthesized by Applied Biosystems (Thermo Fisher Scientific, Waltham, MA, USA).

**Table 1:** The DNA sequence of oligonucleotides used in this study.

Primers and probe	DNA sequences of oligonucleotides	References
Porcine-97bp-F	5’- CGTAGGTGCACAGTAGGTCTGAC - 3’	[12, 30]
Porcine-97bp-R	5’- GGCCAGACTGGGGACATG - 3’	
Porcine-97bp-P	5’- [FAM]-CCAGGTCGGGGAGTC – [NFQ-MGB] – 3’	

FAM, 6-Carboxyfluorescein; MGB, Minor Groove Binder (non-fluorescent chromophore)

The primer-probe set was expected to generate a 96 bp amplicon length by PCR amplification. The primer-probe attachment map is shown in S2 Fig.

### Plasmid copy number preparation

To calculate the plasmid DNA copy number, 4 μg of lyophilized pUC57 recombinant was dissolved in 40 μL nuclease-free water (NFW) to obtain 100 ng/μL. The tube was spun-down and maintained at room temperature for 5 min. A 100 ng/μL stock concentration of pUC57 was then obtained, which was referred to as the stock plasmid. The tube was vortexed and spun for 5 s each to ensure that the solution homogenized and collected at the bottom of the tube. A 25× dilution was performed to obtain a working solution of 4 ng/μL with a total volume of 250 μL. Estimated plasmid copy numbers were calculated based on Avogadro’s number and the molecular weights of each nucleotide using the formula (NA × C)/MW, where NA is the Avogadro constant expressed in mol^-1^, C is the concentration expressed in g/μL, and MW is the molecular weight expressed in g/mol.

### qPCR procedure

qPCR amplification was performed using the CFX Opus Real-Time PCR System (Bio-Rad, Laboratories, Hercules, CA, USA), except for the inter-assay analysis that used six different qPCR systems. The optimized 25 μL reaction mixture contained 12.5 μL of TaqPath^™^ ProAmp^™^ master mix (Applied Biosystems), 5 μL of DNA template, 1 μL of forward/reverse primer (final concentration, 400 nM), 0.5 μL TaqMan^™^ MGB Probe (final concentration, 200 nM), and NFW to the desired final volume. The amplification protocol began with 10 min of 95 °C initial denaturation, followed by 45 cycles of denaturation at 95 °C for 15 s, and an annealing-extension at 60 °C for 1 min. The non-template control was included in each run, and all performed runs were measured with three replications. Fluorescent signals were collected at the annealing-extension stage. Data was obtained using Bio-Rad CFX Maestro V2.0 software (Bio-Rad).

### Droplet digital PCR (ddPCR) procedure

All required reagents were thawed prior to use. Each reaction was amplified with three replicates. According to the manufacturer’s protocol, the ddPCR reaction mixtures were prepared using 10 μL of ddPCR^™^ Supermix for Probes (No dUTP) (Bio-Rad), 4 μL of DNA template, 1.8 μL of forward/reverse primer (final concentration, 900 nM), 0.5 μL of TaqMan^™^ MGB Probes (final concentration 250 nM), 1 μL (5 units) of restriction enzyme *Hind*III (Promega, Madison, WI, USA), and 0.9 μL of nuclease free-water (Thermo Fisher Scientific). A total of 20 μL of each reaction solution was used for droplet preparation using a QX200^™^ Droplet Generator (Bio-Rad). Approximately 20 000 droplets were generated from each reaction by random distribution. The generated droplets were transferred and subjected to PCR amplification using a T100 Thermal Cycler (Bio-Rad). The amplification protocol was performed with an initial denaturation at 95 °C for 10 min, followed by 40 cycles of denaturation at 94 °C for 30 s, and annealing-extension at 60 °C for 60 s. Enzyme inactivation was performed at 98 °C for 10 min and a final incubation at 4 °C. The entire PCR protocol was conducted at a ramp rate of 2 °C/s. Once the amplification was complete, the target DNA was quantified using a QX200^™^ ddPCR Droplet Reader (Bio-Rad) by following the Poisson law for analysis of DNA distribution copies.

### Assay linearity, limit of detection (LOD), and limit of quantitation (LOQ)

The recombinant pUC57 plasmid was used to evaluate the linearity, LOD, and LOQ for both the ddPCR and qPCR systems. Serial dilutions were prepared for the target plasmid as follows: 10^5^, 10^4^, 10^3^, 10^2^, and 10^1^ copies for the linearity test; and 10^5^, 10^4^, 10^3^, 10^2^, 50, 25, 10, 5, and 1 copy for the LOQ and LOD tests. The dilution series was produced using NFW as a buffer (Thermo Fisher Scientific). Serial dilution was performed in three replicates, and a non-template control was included for each run. Target amplification and quantification were determined following the ddPCR and qPCR protocols described in the droplet digital PCR (ddPCR) procedure section.

### Assessment of robustness and inter-assay variability of the qPCR systems

The Laboratory of National Measurement Standards of Biology, which is part of The National Standardization Agency of Indonesia, organized the robustness and inter-assay variability experiments. A total of thirteen laboratories consisting of six university laboratories, three private companies, and four government institutions contributed to the halal testing and supervising. The participant laboratories each received pUC57 containing 10^4^ copies/reaction, which was the concentration used for the evaluations. Inter-assay variability was performed independently five times with three replicates using the CFX Opus Real-Time PCR System (Bio-Rad). The coefficient of variation (CV) was determined by calculating the standard deviation (SD).

Robustness was evaluated using six qPCR models: the CFX Opus Real-time PCR System (Bio-Rad), QuantStudio 5 Real-Time PCR (Thermo Fisher Scientific), qTower3 G Real-Time PCR (Analytik Jena, Jena, Germany), QuantStudio 3 Applied Biosystem (Thermo Fisher Scientific), Rotor-Gene Q Series (Qiagen, Hilden, Germany), and PowerAmp96DX (Kogene Biotech, Seoul, Korea). The evaluation was performed on a pUC57 with a concentration of 10^4^ copies and three replications. The mean, SD, and CV were acquired based on the C_q_ value of each amplification curve.

### Assay performance with a multiple-sample analysis

The defined assay and protocol were developed for porcine detection using several types of samples to evaluate the effectiveness of the assay in a routine inspection. Samples of powdered pork muscle, wild boar meat, porcine floss, powdered gelatin pork skin, and capsules were used in this study. The standard sampling procedure adequately monitored all sample types. The sample variation and extraction kits used in the DNA extraction are shown in Table 2.

**Table 2:** Sample and extraction kit used in this study.

No	Sample types	Resource	Extraction kit
1	Powder Pork Muscle	Sigma-Aldrich	DNeasy mericon food kit (Qiagen, USA)
2	Wild boar meat	Local market in Jakarta	DNeasy mericon food kit (Qiagen, USA)
3	Porcine floss	Local market in Jakarta	EasyFast Pharma I extraction kit (Progenus, Belgium)
4	Powder gelatin pork skin	Sigma-Aldrich	EasyFast Pharma I extraction kit (Progenus, Belgium)
6	Capsule	Local market Jakarta	EasyFast Pharma I extraction kit (Progenus, Belgium)

### Data analysis

The data was analyzed using specific instrument software. Real-time PCR was analyzed using CFX Maestro 2.0 software (Bio-Rad), and the resultant amplification curve and C_q_ values were used for further analysis. Data from ddPCR were evaluated using QuantaSoft^™^ Version 1.7.4 (Bio-Rad). Positive droplets in the experiment indicated that target DNA existed in the amplification process, whereas negative droplets signified no target DNA in the amplification process. The clusters of positive and negative droplets were separated through a threshold line immediately above the negative droplets. The acceptance threshold for total droplets per well was above 10 000 and suitable for the data analysis. The DNA copy number for each reaction was obtained directly from the ddPCR software using the Poisson error algorithm and the total error from a random distribution. The ddPCR linear regression was determined by plotting the value of the measured copy number and the assigned copy number using Microsoft Excel (Microsoft, Redmond, WA, USA).

## Results

### Assessment of dilution accuracy and absolute quantification

**Fig 2:**
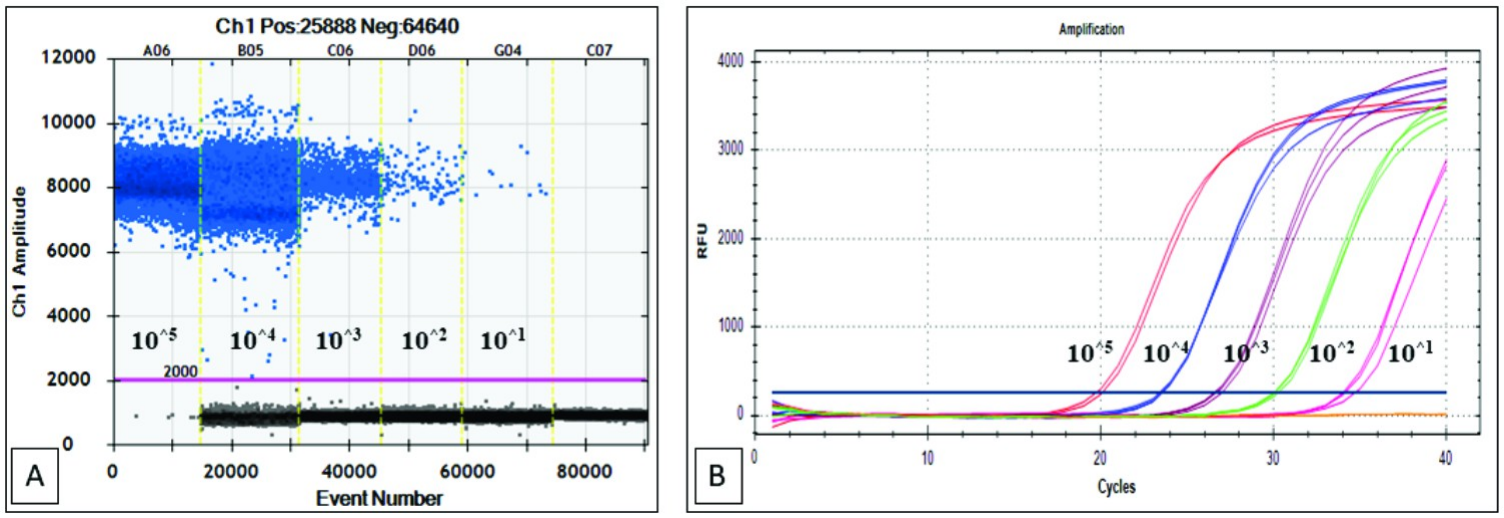
Absolute quantification of the serially diluted recombinant plasmid. A representative 1D amplitude plot of ddPCR reactions (A) and amplification curve of qPCR (B). ddPCR threshold is shown by a pink line that divides the results as positive (above the threshold) and negative droplets (under the threshold). The vertical yellow line divides the ddPCR reactions with different diluted targets. A blue-dark line with the position shows qPCR threshold is in the middle of the logarithmic phase of the amplification curve.

In this study, the target-containing pUC57 was the amplification target for all tests except the multiple-sample assessments. The plasmid was determined based on the Avogadro approach, subjected to 10-fold serial dilution, and quantified by the ddPCR method. Thus, the accuracy of the dilution was tested since the quality of sample dilution was critical for the result. The qPCR results showed a consistent difference in Cq values between each dilution serial of approximately 3.56 (Table 3, Fig 2B), which was subsequently validated using a QX200^™^ ddPCR Droplet Reader (Bio-Rad) by measuring the absolute number of target DNA copies from each dilution serial. The results were expressed by plasmid copy number using Avogadro’s number (copies/reaction) and by plasmid concentration expressed in copies/μL (Table 3). The amplitude fluorescence data from the sample showed that no plasmid copy number was detected in the non-template control (NTC) and that cluster patterns were well separated for both positive and negative droplets (Fig 2A).

**Table 3:** Comparison of droplet digital PCR and qPCR systems using serially diluted pUC57 recombinant plasmid.

Assigned copy numbers per reaction	QPCR	ddPCR	
	Mean Cq value	Measured mean concentration (copies/reaction)	Measured mean concentration (copies/μL)
4.8 x 10^5^	19.94	1.3 x 10^5^	6,500
4.8 x 10^4^	23.39	1.5 x 10^4^	787
4.8 x 10^4^	26.52	1.3 x 10^3^	66.8
4.8 x 10^2^	29.98	2.1 x 10^2^	10.5
4.8 x 10^1^	34.18	1.8 x 10^1^	0.9
H_2_0	ND	ND	ND

### Assessments of linearity and the limit of detection

**Fig 3:**
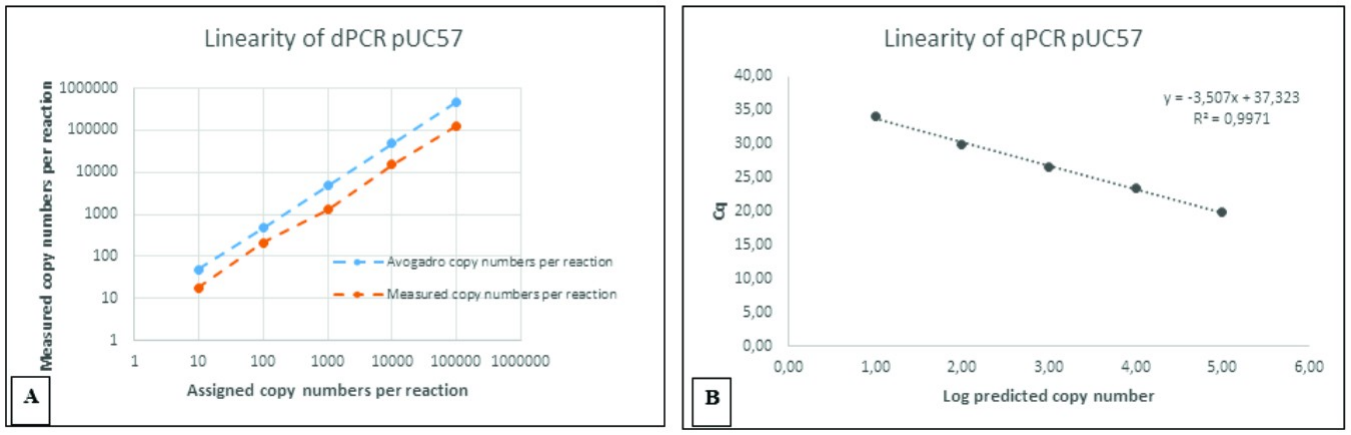
Standard curves and quantification correlation were constructed by (A) ddPCR and (B) qPCR systems for linearity assessment. A quantification correlation was obtained by measuring copy number per reaction against the assigned copy number per reaction **(A)**. A quantification correlation was obtained by Cq value against log starting concentration for QPCR **(B)**. The correlation coefficient (*R*^*2*^) assay for recombinant plasmid pUC57 in ddPCR and qPCR were 0,9998 and 0,9971, respectively.

**Fig 4:**
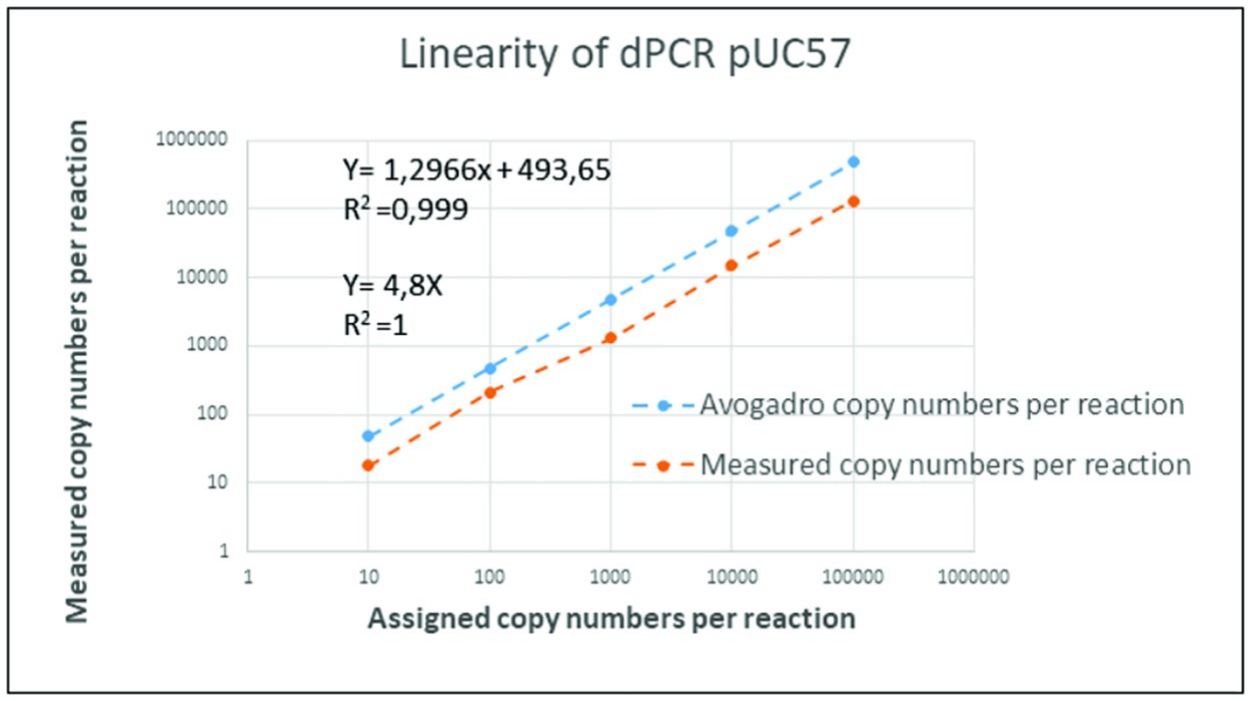
The linearity and quantification correlation of ddPCR between measured copy number per reaction and assigned copy number per reaction.

A series of serially diluted plasmids was prepared similarly for the ddPCR and qPCR assays. Each reaction was performed using the defined protocol for assessing linear relationships and detection limits. The data generated from both qPCR and droplet digital PCR systems exhibited excellent precision, which was the expected result (Fig 3). The linearity of the reaction based on linear regression analysis resulted in correlation coefficient R^2^ values of 09971 and 0.9998 for qPCR and ddPCR, respectively (Fig 4); therefore, the linearity was within the required range of ≥ 0.98 [31].

**Fig 5:**
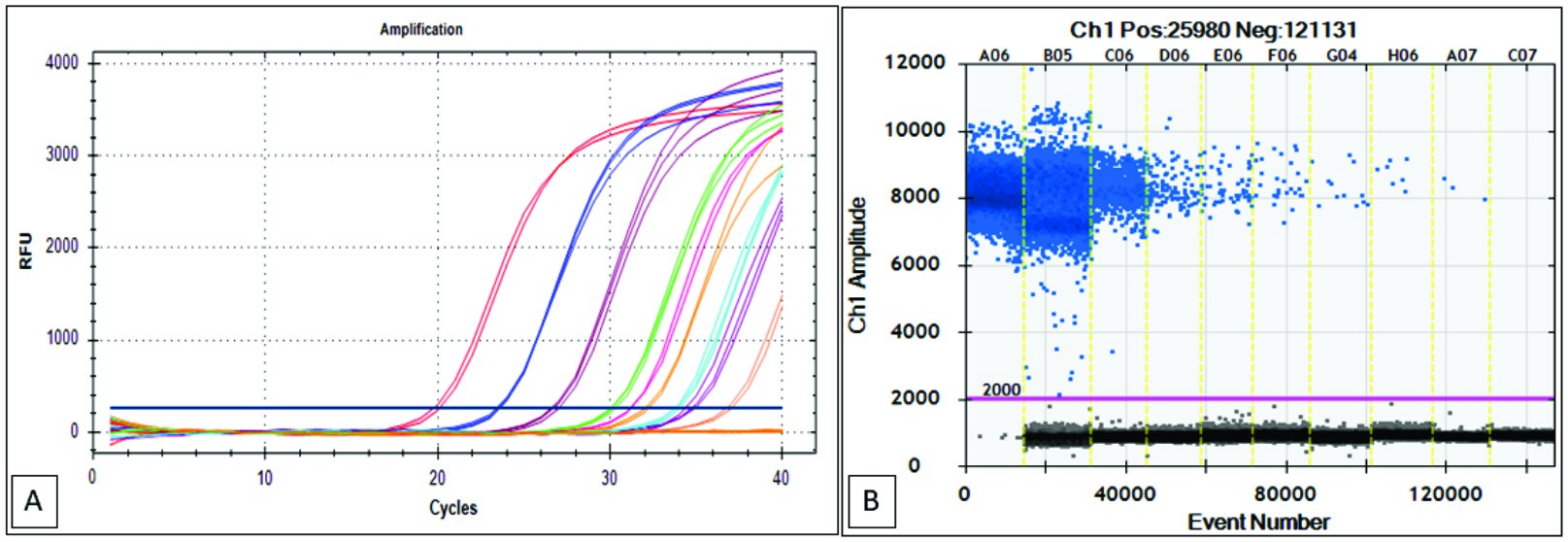
Quantification of serially diluted recombinant plasmid using qPCR and ddPCR systems. A representative LOD and LOQ amplification curve of qPCR **(A)** and 1D amplitude plot of ddPCR reactions **(B)**.

The decision for the regulation of porcine detection should be based on absolute positive or negative results. Thus, it is essential to develop validated protocols that consistently detect the lowest levels of the target DNA possible. Based on the number of positive droplets, the limit of detection for the defined condition using ddPCR was established as one copy number/reaction, while that for the qPCR was five copy numbers/reaction (Fig 5 and Table 4).

**Table 4:** Comparing the limit of detection and quantification of pUC57 recombinant plasmid using qPCR and ddPCR systems. Quantification using ddPCR was shown in copy number due to its capability to perform absolute quantification.

Assigned copy numbers per reaction	qPCR	ddPCR	
	Mean Cq value	Measured mean concentration (copies/reaction)	Measured mean concentration (copies/μL)
Ten ^5	20.03	1.8 x 10^5^	9200
10^4	23.65	2.0 x 10^4^	1010
10^3	26.76	2.0 x 10^3^	102
10^2	30.16	2.0 x 10^2^	10.1
~50	32.23	48	2.4
~25	34.18	18	0.9
~10	34.96	10.6	0.53
~5	37.00	5	0.25
~1	ND	1.8	0.09
H_2_O	ND	ND	ND

### Assessment of inter-assay variability and robustness

**Fig 6:**
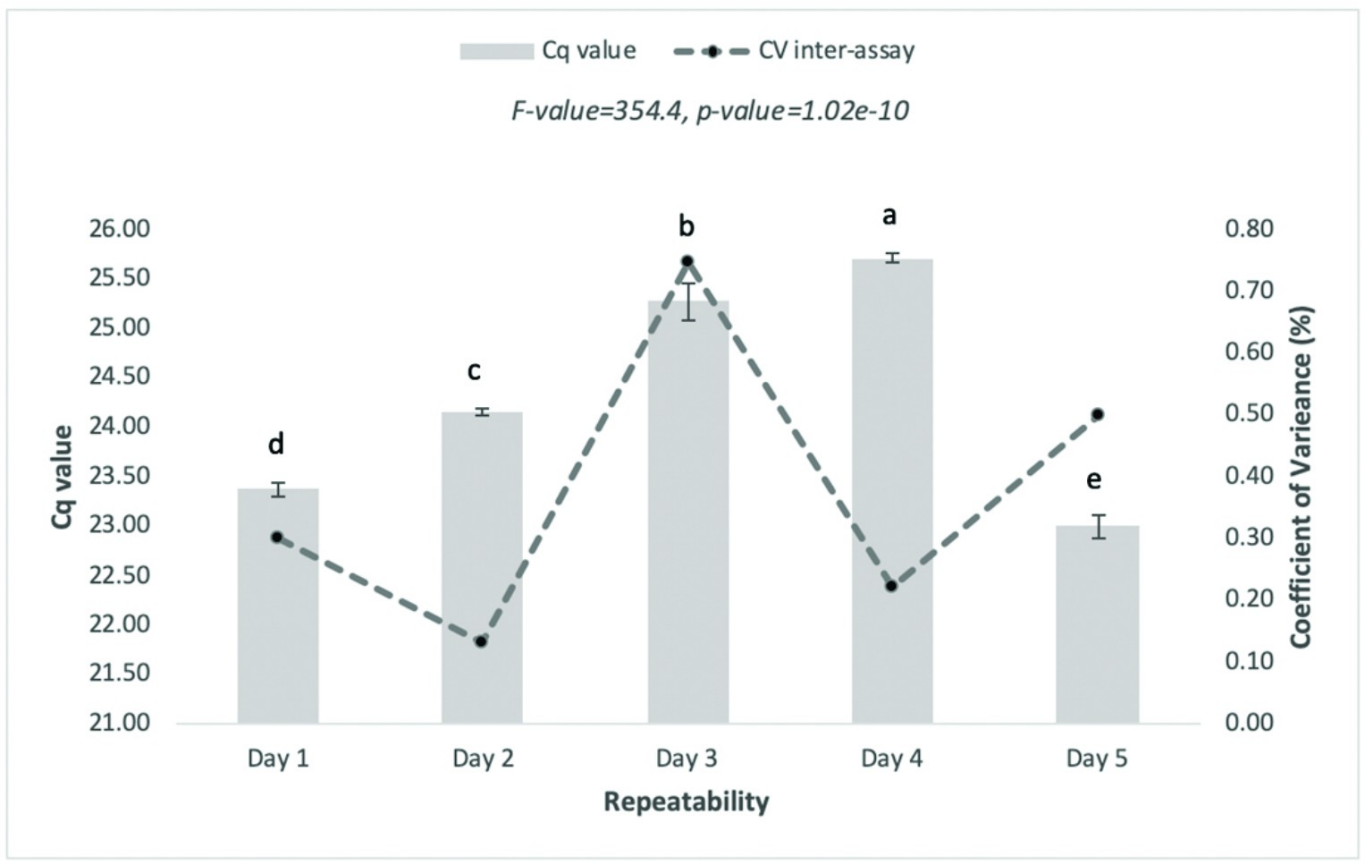
Results of inter-assay variability performed by qPCR systems (shown in CV%). The assessment was carried out by twelve different laboratories using the pUC57 plasmid with a concentration of 10^4^ copies/reaction using CFX Real-Time PCR Systems (Bio-Rad).

**Fig 7:**
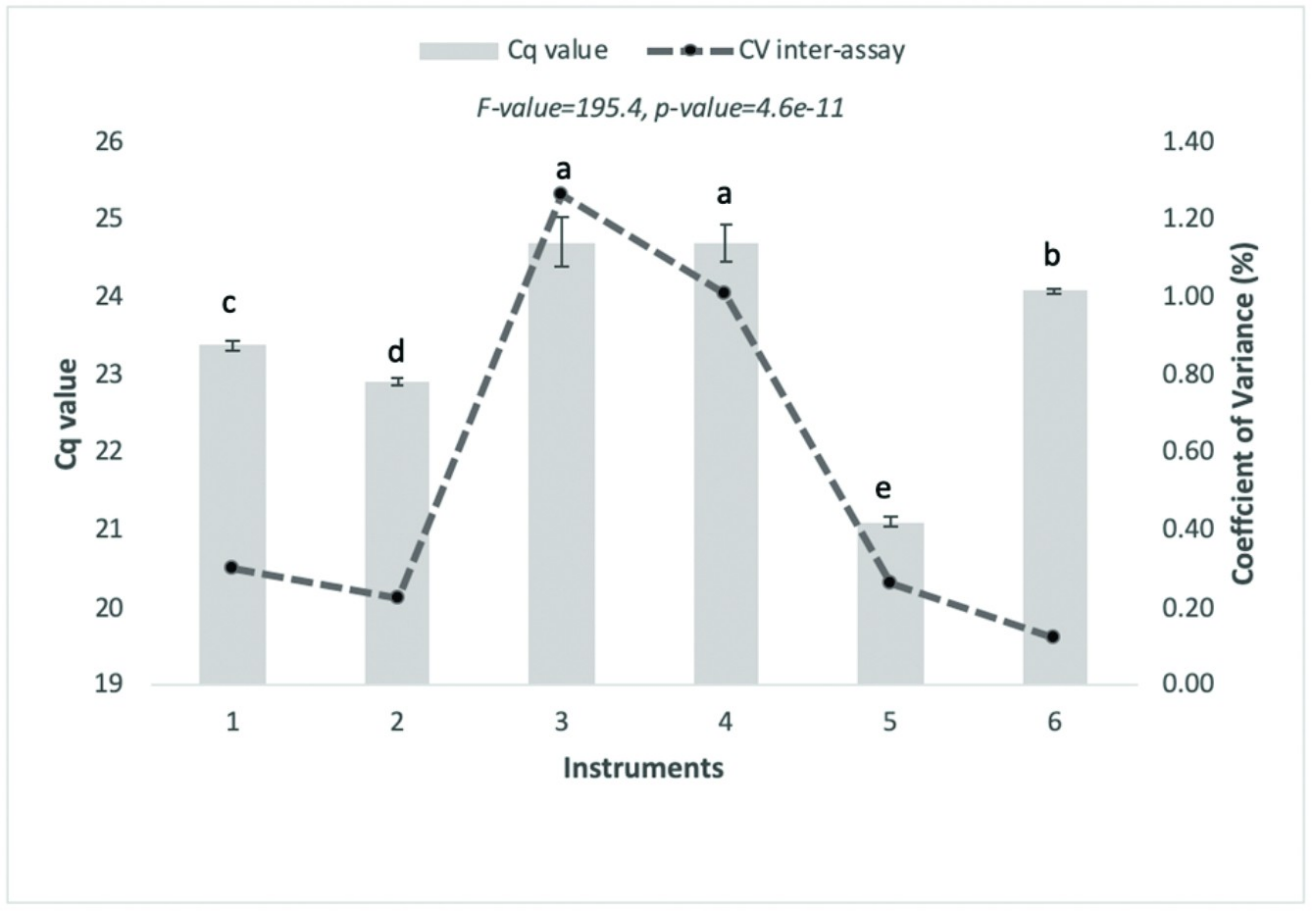
Results of robustness test performed by qPCR systems (shown in CV%). The assessment was carried out by various qPCR models using the pUC57 plasmid with a concentration of 10^4^ copies/reaction.

Assay reproducibility and robustness analyses were performed using a pUC57 of 10^4^ copies/μL. The inter-assay variability and robustness assessments were determined based on the Ct values obtained from triplicate samples within the five-day independent run (inter-assay) employing six kinds of qPCRs (Robustness). Coefficients of variance (CV) for the inter-assays ranged from 0.1% to 0.7%, whereas those for robustness ranged from 0.1% to 1.2%. Detailed information on the inter-assay and robustness results is provided in Figs 6 and 7.

### Assay performance on multiple-sample analysis using qPCR

The performance of the beta-actin gene was only tested with raw meat matrices [12]. Further testing was conducted for this assay using different matrices and processing levels. All tested samples were confirmed positive and contained traces of porcine DNA. The Cq value gradually diminished from the more highly processed samples (gelatin, capsule, and porcine floss) to the raw meat (Table 5). A higher Cq value indicates less copy DNA because the highly processed food contained DNA that was degraded into short fragments [32]. Multiple-sample analysis was important for halal authentication because of the diversity of porcine secondary products used in most food and pharmaceutical products.

**Table 5:** Assay performance on various sample analyses using qPCR.

No	Samples type	Status	Cq of Amplification curve					Standard Deviation (SD)	CV (%)	Confirmed positivity rate (%)
			R1	R2	R3	R4	Mean			
1	Powder pork muscle	Positive	25.76	26.33	25.08	23.74	25.23	0.63	2.48	100
2	Wild boar meat	Positive	20.98	20.91	20.48	20.26	20.66	0.35	1.67	100
3	Porcine floss	Positive	31.19	30.73	31.11	30.99	31.01	0.20	0.65	100
4	Powder gelatin pork skin	Positive	36.96	37.04	37.62	36.28	36.98	0.55	1.48	100
5	Capsule	Positive	36.23	36.76	37.1	37.22	36.83	0.44	1.19	100
6	C(+)*10^4^ pUC57	Positive	23.47	23.33	23.41	23.25	23.365	0.07	0.30	
7	C(-)** NTC		ND	ND	ND	ND	ND	0	0	

• C(+)*10^4^ pUC57 = positive control, containing pUC57 plasmid, 10^4^

• C(-)** NTC = negative control, no template control

## Discussion

Currently, quantitative polymerase chain reaction (qPCR) is widely used for the detection and quantification of porcine matrices in food and drugs, and several publications have specifically focused on qPCR assay and protocols for porcine detection. qPCR produces acceptable precision, development and optimization methods, targets, and reactions for porcine detection and can be conducted independently by companies and laboratories based on general guidelines or scientific publication numbers. However, this approach may lead to varying results due to differences in tools, methods, targets, and human resources. Thus, there is a need to standardize the qPCR protocol for national-wide application.

Droplet digital PCR, a relatively new technique, is promising for low-level DNA detection and absolute quantification. This method is less affected by inhibitory matrices [21], less susceptible to interference [33], more accurate for meat quantification in mixtures, and exhibits high stability and repeatability [34]. However, the necessary materials and instrumentation costs are high compared to those required for qPCR [34]. This study described the development of a qPCR standard protocol using an artificial recombinant plasmid pUC57 as a standard control for porcine DNA detection and reliability and traceability determination of absolute quantification, which was then compared to the ddPCR technique. The qPCR results showed a consistent difference in Cq values between each dilution serial of approximately 3.56. The qPCR results were subsequently validated using a QX200^™^ ddPCR Droplet Reader (Bio-Rad) by measuring an absolute number of copies of the target DNA from each dilution serial. The results were expressed by plasmid copy number using Avogadro’s number (copies/reaction) and by plasmid concentration expressed in copies/μL (Table 3). The amplitude fluorescence data detected no plasmid copy number in the NTC, and cluster patterns were well separated for both positive and negative droplets (Fig 2A).

Several parameters are involved in determining the performance of an in-house protocol, and of these, the limit of detection (LOD) can be the most important [35]. The definition of LOD differs among the perspectives of the literature and standard organizations [36, 37]. A previous study [38] indicated that the limit of detection is "the lowest amount of analyte (measured) in a sample that can be detected with (stated) probability, although perhaps not quantified as an exact value."

Based on the number of positive droplets, the detection limit for the defined conditions with the ddPCR was one copy number/reaction. This confirms that the ddPCR technology provided a high consistency and sensitivity for the detection of low levels of DNA. In contrast, the qPCR results revealed a detection limit of five copy numbers/reaction with a Cq value of 37 from 40 total cycles (Table 4). This result was slightly less sensitive than the ddPCR result but still reliable and reasonable. The Cq value corresponding to the detection limit is suitable for routine inspection and decision-making systems.

The data proved the high precision and efficiency of the defined assay and protocols used in this study, with a similar level of performance for the qPCR and ddPCR technologies. However, the reaction was conducted in low or absent levels of matrix or background contaminants and the sensitivity could be determined by other factors, such as the sample homogeneity, DNA extraction efficiency, matrices, and target DNA concentration [39].

Beta-actin and other single-copy genes are less effective than mitochondrial DNA (mtDNA) for assessing the authentication of porcine testing, possibly because mtDNA is abundant in cells, thereby increasing its amplification probability. However, our testing found that beta-actin was desirable even in low-quantity DNA samples such as those of gelatin and capsules. In addition, the large amounts of mtDNA can be a disadvantage for the development of validation standards due to quantity uncertainty.

Furthermore, inter-assay and robustness tests were performed independently by twelve participating laboratories. We found that a CV value of less than 10% (inter-assay CV and 15% inter-assay CV) was regarded as acceptable [40]. The participating laboratories were distributed throughout Indonesia and had different instrumentation, laboratory standards, and practical human resources. The reports showed no apparent problems in relation to the experimental protocols, indicating the applicability of the assays. The experimental evaluation of inter-assay and assessment robustness showed that the values obtained were acceptable, indicating that the assay is reproducible and robust. The results indicated that the absolute quantification and linearity assessment had high precision with R2 values of 0.9971 and 0.9998 for qPCR and ddPCR, respectively. Moreover, the evaluation of this method by multiple samples and inter-participatory testing showed high sensitivity, broad applicability, and robustness.

## Conclusion

Porcine adulteration has been gaining interest in food authentication and is currently a concern for halal and kosher food verification. DNA-based methods, especially qPCR, have been widely used for the identification and quantification of various food samples. However, certain limitations exist that must be resolved to develop a well-established protocol in this area. Digital PCR (dPCR) is a newly developed method for absolute quantification of target DNA copy numbers that has been proposed to overcome these limitations. This experimental study is the first to perform a direct comparison between qPCR and dPCR porcine detection methods using an artificial recombinant plasmid to eliminate any sample matrix biases. In general, dPCR methods proved more precise at concentrations lower than 5 copy numbers. However, at higher concentrations, the methods revealed comparable linearity and detection limits. The data derived from this experiment could be considered by regulatory authorities toward the development of a national protocol for Indonesia. The use of dPCR may not be feasible for routine general sample testing due to the cost restraints, but it has potential as a routine inspection and quality control method for instrument performance, kit, and interlaboratory-user capabilities in national-wide inspections. This result could also encourage the development of a specific protocol for low-concentration samples using a recombinant plasmid as a qPCR reference material with Cq threshold determination. However, to achieve a well-established protocol, the research should be conducted in a manner that includes all factors and conditions that could occur during routine testing. Thus, additional research is needed to expand the food matrix and concentration ranges, as well as to define the specificity and measurement uncertainty.

## Supporting information

S1 Fig Plasmid map and DNA sequence annotation of pUC57 with partial porcine beta actin insert (ACTB) gene. (TIF)

S2 Fig Map of forward/reverse/probe attachment position on partial beta actin gene (ACTB) which produces a 97 bp amplification product. (TIF)

## References

[1] Codex Alimentarius Commission. Discussion Paper On Consideration Of Emerging Issues And Future Directions For The Work Of The Codex Committee On Food Import And Export Inspection And Certification Systems [Internet]. Australia; 2018 Oct [cited 2022 Aug 16] p. 1–13. https://www.fao.org/fao-who-codexalimentarius/sh-proxy/en/?lnk=1&url=https%253A%252F%252Fworkspace.fao.org%252Fsites%252Fcodex%252FMeetings%252FCX-733-24%252FWorking%2BDocuments%252Ffc24_08e.pdf

[2] Tanabe S, Hase M, Yano T, Sato M, Fujimura T, Akiyama H. A Real-Time Quantitative PCR Detection Method for Pork, Chicken, Beef, Mutton, and Horseflesh in Foods. Biosci Biotechnol Biochem. 2007;71(12):3131–5. doi: 10.1271/bbb.70683 18071237

[3] European Commission. Commission Directive 2002/86/EC [Internet]. L/305/19 Jun 11, 2002. https://eur-lex.europa.eu/LexUriServ/LexUriServ.do?uri=OJ:L:2002:305:0019:0019:EN:PDF

[4] FAO. Food fraud–Intention, detection and management: Food safety technical toolkit for Asia and the Pacific [Internet]. Bangkok, Thailand: FAO; 2021 [cited 2022 Aug 16]. 44 p. https://www.fao.org/documents/card/en/c/cb2863en/

[5] Jalil NSA, Tawde AV, Zito S, Sinclair M, Fryer C, Idrus Z, et al. Attitudes of the public towards halal food and associated animal welfare issues in two countries with predominantly Muslim and non-Muslim populations. PLOS ONE. 2018 Oct 31;13(10):e0204094. doi: 10.1371/journal.pone.0204094 30379818 PMC6209144

[6] Yusop MHM, Bakar MFA. Review on halal forensic: a focus on DNA-based methods for pork authentication. Food Res. 2020 Nov 8;4(6):2347–54.

[7] Nhari RMHR, Hanish I, Mokhtar NFK, Hamid M, El Sheikha A f. Authentication approach using enzyme-linked immunosorbent assay for detection of porcine substances. Qual Assur Saf Crops Foods. 2019 Sep 11;11(5):449–57.

[8] El Sheikha AF, Mokhtar NFK, Amie C, Lamasudin DU, Isa NM, Mustafa S. Authentication technologies using DNA-based approaches for meats and halal meats determination. Food Biotechnol. 2017;31(4):281–315.

[9] Sentandreu MA, Fraser PD, Halket J, Patel R, Bramley PM. A Proteomic-Based Approach for Detection of Chicken in Meat Mixes. J Proteome Res. 2010 Jul 2;9(7):3374–83. doi: 10.1021/pr9008942 20433202

[10] El Sheikha AF. DNAFoil, a novel technology for the rapid detection of food pathogens: Preliminary validation on Salmonella and Listeria monocytogenes. Ital J Food Sci. 2021 Apr 19;33(SP1):43–54.

[11] Kesmen Z, Yetiman AE, Şahin F, Yetim H. Detection of Chicken and Turkey Meat in Meat Mixtures by Using Real-Time PCR Assays. J Food Sci. 2012;77(2):C167–73. doi: 10.1111/j.1750-3841.2011.02536.x 22309374

[12] Wang Q, Cai Y, He Y, Yang L, Pan L. Collaborative ring trial of two real-time PCR assays for the detection of porcine- and chicken-derived material in meat products. PLoS ONE. 2018;13(10):e0206609. doi: 10.1371/journal.pone.0206609 30372489 PMC6205609

[13] Perestam AT, Fujisaki KK, Nava O, Hellberg RS. Comparison of real-time PCR and ELISA-based methods for the detection of beef and pork in processed meat products. Food Control. 2017 Jan 1;71:346–52.

[14] Tang H, Cai Q, Li H, Hu P. Comparison of droplet digital PCR to real-time PCR for quantification of hepatitis B virus DNA. Biosci Biotechnol Biochem. 2016 Nov;80(11):2159–64. doi: 10.1080/09168451.2016.1196576 27310131

[15] Taylor SC, Laperriere G, Germain H. Droplet Digital PCR versus qPCR for gene expression analysis with low abundant targets: from variable nonsense to publication quality data. Sci Rep. 2017 May 25;7(1):2409. doi: 10.1038/s41598-017-02217-x 28546538 PMC5445070

[16] Zhao Y, Xia Q, Yin Y, Wang Z. Comparison of Droplet Digital PCR and Quantitative PCR Assays for Quantitative Detection of Xanthomonas citri Subsp. citri. PLOS ONE. 2016 Jul 18;11(7):e0159004. doi: 10.1371/journal.pone.0159004 27427975 PMC4948846

[17] Gong J, Yang C, Khafipour E. Molecular and “Omics”Techniques for Studying Gut Microbiota Relevant to Food Animal Production. In: Sheikha AFE, Levin RE, Xu J, editors. Molecular Techniques in Food Biology: Safety, Biotechnology, Authenticity and Traceability. John Wiley & Sons; 2018. p. 71–94.

[18] Pinheiro LB, Coleman VA, Hindson CM, Herrmann J, Hindson BJ, Bhat S, et al. Evaluation of a Droplet Digital Polymerase Chain Reaction Format for DNA Copy Number Quantification. Anal Chem. 2012 Jan 17;84(2):1003–11. doi: 10.1021/ac202578x 22122760 PMC3260738

[19] Hindson CM, Chevillet JR, Briggs HA, Gallichotte EN, Ruf IK, Hindson BJ, et al. Absolute quantification by droplet digital PCR versus analog real-time PCR. Nat Methods. 2013 Oct;10(10):1003–5. doi: 10.1038/nmeth.2633 23995387 PMC4118677

[20] Cai Y, He Y, Lv R, Chen H, Wang Q, Pan L. Detection and quantification of beef and pork materials in meat products by duplex droplet digital PCR. PLoS ONE. 2017;12(8):1–12. doi: 10.1371/journal.pone.0181949 28771608 PMC5542382

[21] Hoshino T, Inagaki F. Molecular quantification of environmental DNA using microfluidics and digital PCR. Syst Appl Microbiol. 2012 Sep 1;35(6):390–5. doi: 10.1016/j.syapm.2012.06.006 22840894

[22] Hindson BJ, Ness KD, Masquelier DA, Belgrader P, Heredia NJ, Makarewicz AJ, et al. High-Throughput Droplet Digital PCR System for Absolute Quantitation of DNA Copy Number. Anal Chem. 2011 Nov 15;83(22):8604–10. doi: 10.1021/ac202028g 22035192 PMC3216358

[23] Bogožalec Košir A, Demšar T, Štebih D, Žel J, Milavec M. Digital PCR as an effective tool for GMO quantification in complex matrices. Food Chem. 2019 Oct 1;294:73–8. doi: 10.1016/j.foodchem.2019.05.029 31126507

[24] Ricchi M, Bertasio C, Boniotti MB, Vicari N, Russo S, Tilola M, et al. Comparison among the Quantification of Bacterial Pathogens by qPCR, dPCR, and Cultural Methods. Front Microbiol [Internet]. 2017 [cited 2022 Aug 16];8. Available from: https://www.frontiersin.org/articles/10.3389/fmicb.2017.01174 28702010 10.3389/fmicb.2017.01174 PMC5487435

[25] Floren C, Wiedemann I, Brenig B, Schütz E, Beck J. Species identification and quantification in meat and meat products using droplet digital PCR (ddPCR). Food Chem. 2015 Apr 15;173:1054–8. doi: 10.1016/j.foodchem.2014.10.138 25466124

[26] Shehata HR, Li J, Chen S, Redda H, Cheng S, Tabujara N, et al. Droplet digital polymerase chain reaction (ddPCR) assays integrated with an internal control for quantification of bovine, porcine, chicken and turkey species in food and feed. PLOS ONE. 2017 Aug 10;12(8):e0182872. doi: 10.1371/journal.pone.0182872 28796824 PMC5552122

[27] Alhamid G, Tombuloglu H, Rabaan AA, Al-Suhaimi E. SARS-CoV-2 detection methods: A comprehensive review. Saudi J Biol Sci. 2022 Nov 1;29(11):103465. doi: 10.1016/j.sjbs.2022.103465 36186678 PMC9512523

[28] Deprez L, Corbisier P, Kortekaas AM, Mazoua S, Beaz Hidalgo R, Trapmann S, et al. Validation of a digital PCR method for quantification of DNA copy number concentrations by using a certified reference material. Biomol Detect Quantif. 2016 Aug 30;9:29–39. doi: 10.1016/j.bdq.2016.08.002 27617230 PMC5007884

[29] Mohamad NA, El Sheikha AF, Mustafa S, Mokhtar NFK. Comparison of gene nature used in real-time PCR for porcine identification and quantification: A review. Food Res Int. 2013 Jan 1;50(1):330–8.

[30] Cai Y, Li X, Lv R, Yang J, Li J, He Y, et al. Quantitative Analysis of Pork and Chicken Products by Droplet Digital PCR. BioMed Res Int. 2014 Aug 27;2014:e810209. doi: 10.1155/2014/810209 25243184 PMC4163444

[31] Broeders S, Huber I, Grohmann L, Berben G, Taverniers I, Mazzara M, et al. Guidelines for validation of qualitative real-time PCR methods. Trends Food Sci Technol. 2014 Jun 1;37(2):115–26.

[32] Shabani H, Mehdizadeh M, Mousavi SM, Dezfouli EA, Solgi T, Khodaverdi M, et al. Halal authenticity of gelatin using species-specific PCR. Food Chem. 2015 Oct 1;184:203–6. doi: 10.1016/j.foodchem.2015.02.140 25872445

[33] Nakano M, Komatsu J, Matsuura S ichi, Takashima K, Katsura S, Mizuno A. Single-molecule PCR using water-in-oil emulsion. J Biotechnol. 2003 Apr 24;102(2):117–24. doi: 10.1016/s0168-1656(03)00023-3 12697388

[34] Ren J, Deng T, Huang W, Chen Y, Ge Y. A digital PCR method for identifying and quantifying adulteration of meat species in raw and processed food. PLOS ONE. 2017 Mar 20;12(3):e0173567. doi: 10.1371/journal.pone.0173567 28319152 PMC5358868

[35] Bustin SA, Benes V, Garson JA, Hellemans J, Huggett J, Kubista M, et al. The MIQE Guidelines: Minimum Information for Publication of Quantitative Real-Time PCR Experiments. Clin Chem. 2009 Apr 1;55(4):611–22. doi: 10.1373/clinchem.2008.112797 19246619

[36] Morisset D, Štebih D, Milavec M, Gruden K, Žel J. Quantitative Analysis of Food and Feed Samples with Droplet Digital PCR. PLOS ONE. 2013 May 2;8(5):e62583. doi: 10.1371/journal.pone.0062583 23658750 PMC3642186

[37] Villamil C, Calderon MN, Arias MM, Leguizamon JE. Validation of Droplet Digital Polymerase Chain Reaction for Salmonella spp. Quantification. Front Microbiol. 2020 Jul 7;11:1512. doi: 10.3389/fmicb.2020.01512 32733415 PMC7358645

[38] Pierson-Perry JF, Vaks JE, Durham AP. Evaluation of detection capability for clinical laboratory measurement procedures; approved guideline—second edition. Wayne, Pa., U.S.A: CLSI; 2012.

[39] Khairil Mokhtar NF, El Sheikha AF, Azmi NI, Mustafa S. Potential authentication of various meat-based products using simple and efficient DNA extraction method. J Sci Food Agric. 2020 Mar 15;100(4):1687–93. doi: 10.1002/jsfa.10183 31803942

[40] Center for Drug Evaluation and Research, Center for Veterinary Medicine. Bioanalytical Method Validation Guidance for Industry [Internet]. U.S. Food and Drug Administration. FDA; 2020 [cited 2022 Oct 13]. https://www.fda.gov/regulatory-information/search-fda-guidance-documents/bioanalytical-method-validation-guidance-industry

