# Supplementary figures and images for: Droplet digital PCR versus real-time PCR for in-house validation of porcine detection and quantification protocol: An artificial recombinant plasmid approach
Source: PLoS One. 2023 Jul 14;18(7):e0287712. doi: 10.1371/journal.pone.0287712 (PMC10348585; doi:10.1371/journal.pone.0287712)

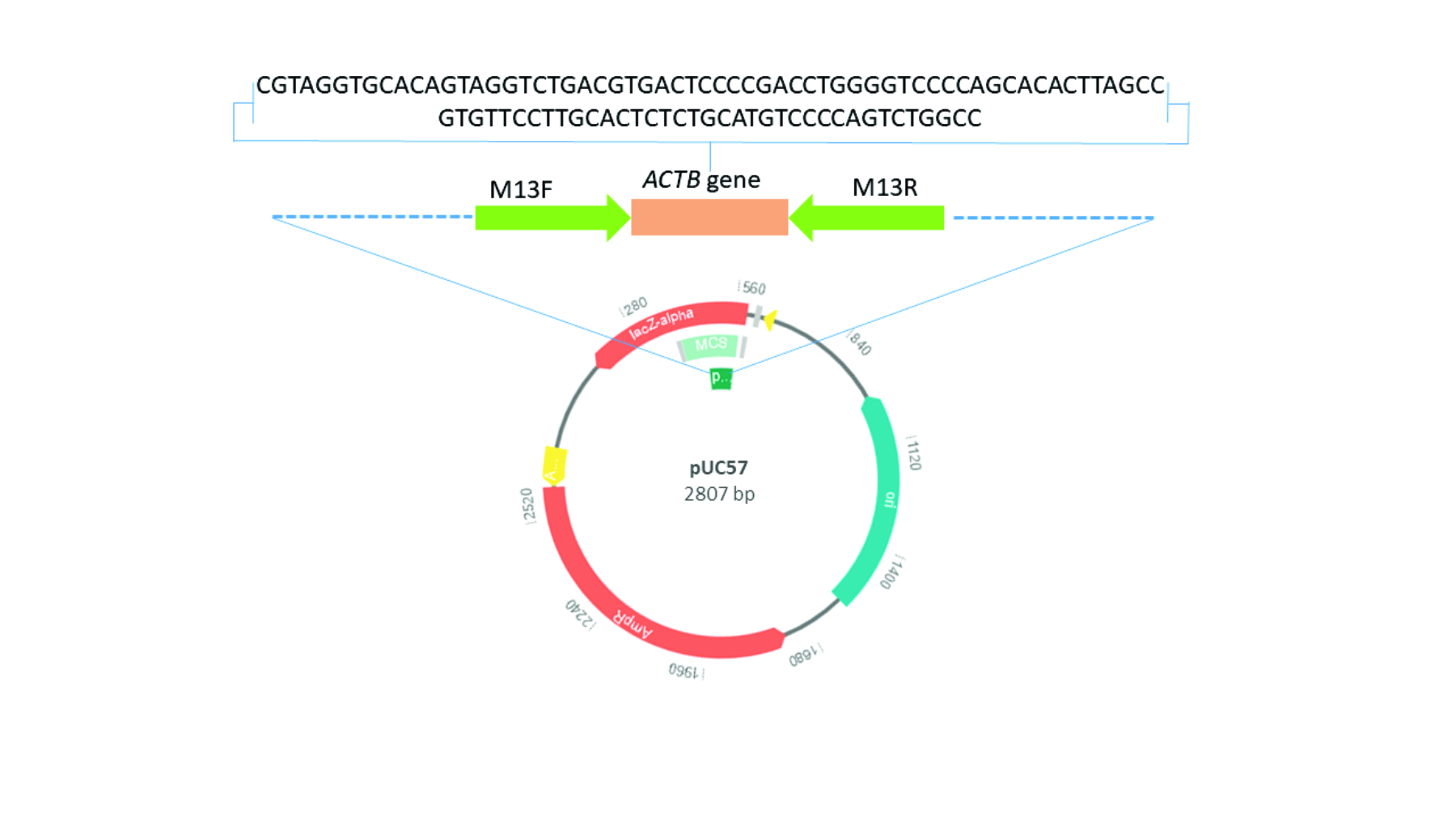

Supplement: S1 Fig — (TIF) [file pone.0287712.s001.tif]

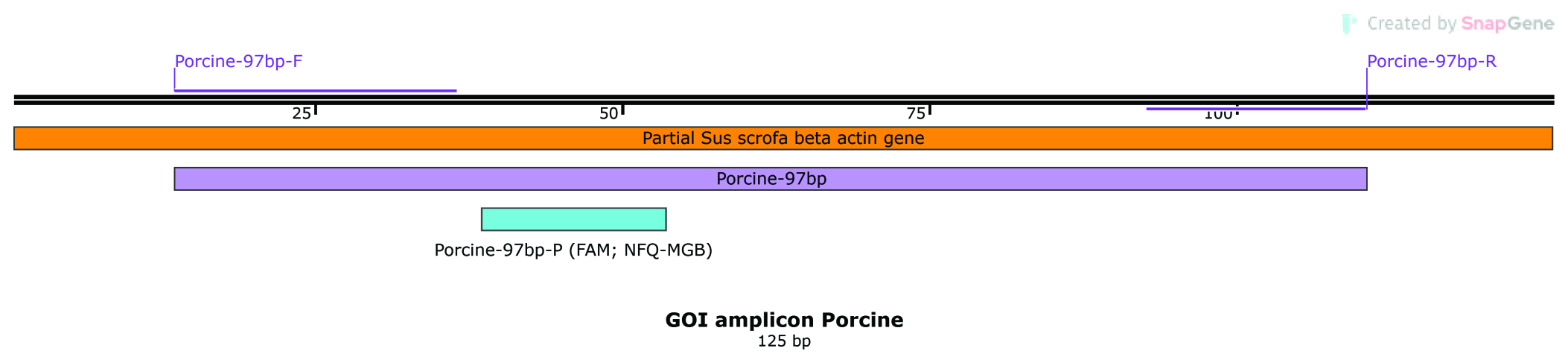

Supplement: S2 Fig — (TIF) [file pone.0287712.s002.tif]
